# The D-score: a metric for interpreting the early development of infants and toddlers across global settings

**DOI:** 10.1136/bmjgh-2019-001724

**Published:** 2019-11-19

**Authors:** Ann M Weber, Marta Rubio-Codina, Susan P Walker, Stef van Buuren, Iris Eekhout, Sally M Grantham-McGregor, Maria Caridad Araujo, Susan M Chang, Lia CH Fernald, Jena Derakhshani Hamadani, Charlotte Hanlon, Simone M Karam, Betsy Lozoff, Lisy Ratsifandrihamanana, Linda Richter, Maureen M Black, Working group members and data contributors, Orazio Attanasio, Gary L Darmstadt, Bernice M Doove, Emanuela Galasso, Pamela Jervis, Girmay Medhin, Ana M B Menezes, Helen Pitchik, Sarah Reynolds, Norbert Schady

**Affiliations:** 1 School of Community Health Sciences, University of Nevada Reno, Reno, Nevada, USA; 2 Department of Pediatrics, Stanford University School of Medicine, Stanford, California, USA; 3 Inter-American Development Bank, Washington, District of Columbia, USA; 4 Caribbean Institute for Health Research, University of the West Indies, Kingston, Jamaica; 5 Netherlands Organization for Applied Scientific Research TNO, Leiden, Netherlands; 6 Methodology & Statistics, Utrecht University, Utrecht, Netherlands; 7 Institute of Child Health, University College London, London, UK; 8 School of Public Health, University of California Berkeley, Berkeley, California, USA; 9 Maternal and Child Health Division, icddr,b, Dhaka, Bangladesh; 10 Institute of Psychiatry, Psychology and Neuroscience, Health Service and Population Research Department, Centre for Global Mental Health, King's College London, London, UK; 11 Department of Psychiatry, WHO Collaborating Centre for Mental Health Research and Capacity Building, School of Medicine, and Centre for Innovative Drug Development and Therapeutic Trials for Africa (CDT-Africa), College of Health Sciences, Addis Ababa University, Addis Ababa, Ethiopia; 12 Department of Pediatrics, Federal University of Rio Grande, Rio Grande, Brazil; 13 Center for Human Growth and Development, University of Michigan, Ann Arbor, Michigan, USA; 14 Centre Médico-Educatif "Les Orchidées Blanches", Antananarivo, Madagascar; 15 Centre of Excellence in Human Development, University of the Witwatersrand, Johannesburg, South Africa; 16 Department of Pediatrics, University of Maryland School of Medicine, Baltimore, Maryland, USA; 17 International Education, RTI International, Research Triangle Park, North Carolina, USA

**Keywords:** child development, global health, psychometrics, item response theory

## Abstract

**Introduction:**

Early childhood development can be described by an underlying latent construct. Global comparisons of children’s development are hindered by the lack of a validated metric that is comparable across cultures and contexts, especially for children under age 3 years. We constructed and validated a new metric, the Developmental Score (D-score), using existing data from 16 longitudinal studies.

**Methods:**

Studies had item-level developmental assessment data for children 0–48 months and longitudinal outcomes at ages >4–18 years, including measures of IQ and receptive vocabulary. Existing data from 11 low-income, middle-income and high-income countries were merged for >36 000 children. Item mapping produced 95 ‘equate groups’ of same-skill items across 12 different assessment instruments. A statistical model was built using the Rasch model with item difficulties constrained to be equal in a subset of equate groups, linking instruments to a common scale, the D-score, a continuous metric with interval-scale properties. D-score-for-age z-scores (DAZ) were evaluated for discriminant, concurrent and predictive validity to outcomes in middle childhood to adolescence.

**Results:**

Concurrent validity of DAZ with original instruments was strong (average *r*=0.71), with few exceptions. In approximately 70% of data rounds collected across studies, DAZ discriminated between children above/below cut-points for low birth weight (<2500 g) and stunting (−2 SD below median height-for-age). DAZ increased significantly with maternal education in 55% of data rounds. Predictive correlations of DAZ with outcomes obtained 2–16 years later were generally between 0.20 and 0.40. Correlations equalled or exceeded those obtained with original instruments despite using an average of 55% fewer items to estimate the D-score.

**Conclusion:**

The D-score metric enables quantitative comparisons of early childhood development across ages and sets the stage for creating simple, low-cost, global-use instruments to facilitate valid cross-national comparisons of early childhood development.

**Summary**

**What is already known?**

- Theories of infant development and empirical evidence support both a universal biological unfolding of stage-based skills as well as individual differences due to genetic, environmental and cultural influences.
- Despite the availability of multiple measures, a common and easily interpretable metric does not exist for making valid international comparisons of children’s development from birth to 3 years.

**What are the new findings?**

- Existing data from 16 longitudinal studies and 11 countries were mathematically linked with an innovative statistical model to construct a common metric, the Developmental Score (D-score), that represents a latent construct for early childhood development.
- The D-score, estimated with an average of 55% fewer items than the original instruments, demonstrated discriminant and concurrent validity and was predictive of outcomes during middle childhood through adolescence.

**What do the findings imply?**

- The D-score’s interval-scale property, with a common unit of measurement across ages, allows for the depiction of a developmental trajectory with increasing age, which can be interpreted similarly to growth trajectories for height and weight.
- The statistical model enables both the estimation of D-scores for existing datasets and the derivation of new instruments, which will allow for valid international comparisons and future construction of global standards for the development of children 0–3 years.

## Introduction

Theories of infant development support both a universal biological unfolding of stage-based skills as well as individual differences due to varying genetic, environmental and cultural influences.^1 2^ Empirical evidence validates these theories by demonstrating that, on average, infants and toddlers achieve major neurodevelopmental milestones in a consistent and ordinal pattern during the first few years of life, regardless of country of origin, while demonstrating within country variability conditional on parental and household disparities.^3 4^ Therefore, from both theoretical and empirical perspectives, childhood development in the first years of life can be described by an underlying latent construct that is relatively invariant across countries, progresses in a predictable sequence and represents domains of motor, language, cognitive and personal-social development. However, we lack a valid and easily interpretable metric that represents a latent construct of early childhood development and would enable global comparisons of child development, just as growth trajectories for height and weight facilitate global comparisons of children’s nutritional status.

There is a long history of testing the emergent developmental skills of infants and toddlers, through direct observation, child’s response to specific tasks and situations, or by caregiver report. As a result, multiple assessment instruments incorporating similar tasks have been developed, most of which are standardised for high-income country populations.^5^ Although some have been used globally, instruments adapted in one setting may not measure the same construct as originally designed, or as adapted in other settings, and may not perform equivalently across countries. As such, global comparisons of scores obtained from adapted instruments may be misleading.

Our goal was to develop and evaluate a metric representing a universal latent construct of early childhood development by leveraging existing data from 16 longitudinal cohorts from 11 countries, gathered using 12 existing instruments. In this paper, we describe the construction of a statistical model using these data to produce the Developmental Score (D-score), an interval-scale metric to express children’s development with a common numerical unit. The D-score facilitates interpretation of children’s abilities across different ages (just as centimetres are used for height), and an age-standardised D-score enables comparisons of children’s development both within and between countries.^6^ We examine discriminant, concurrent and predictive validity of model-derived D-scores for children living in diverse cultural settings. We conclude with a discussion of how the validated D-score metric and model can be used to convert existing data from disparate settings to a common metric and to construct new instruments for global use.

## Methods

### Country and study cohorts

Longitudinal data from 16 cohorts of children (n>36 000) in 11 countries were previously collected as birth cohort studies (Brazil 1 and Brazil 2,^7 8^ Chile 2,^9^ Ethiopia,^10 11^Netherlands 2,^12^and South Africa^13^), instrument validation studies (the Netherlands 1^14^ and Colombia 2^15^), and programme evaluations focused on low-income or undernourished children (Bangladesh,^16^ Chile 1,^17^ China,^18^ Colombia 1,^19^ Ecuador,^20^ Jamaica 1^21^ and Jamaica 2,^22^ and Madagascar^23^). Brazil 2 (Pelotas), Netherlands 2 (Maastricht) and South Africa (Johannesburg-Soweto) cohorts were representative of a city in each country; the Colombia 2 cohort was representative of low-income and low-middle-income groups in Bogota; the Ethiopia cohort was representative of a rural district; and the Chile 2 cohort was representative of the country. Although initially representative of the city of Pelotas, the Brazil 1 data included here were obtained from all low birth weight (<2500 g) children in the cohort and a systematic sample of the remaining cohort members. An Advisory Board was formed that included an investigator from each study with in-depth knowledge about the local context and data collection. Information on these studies, validity and primary analyses were published previously.^6–21^ Table 1 shows an overview of the cohorts.

**Table 1:** Study cohorts: sample sizes, age range and instruments by time of measurement

	Time 1: Early childhood				Time 2: Middle childhood		Time 2: Adolescence		
	Round	N	Age (months)	Instrument*	Age (years;months)	Instrument†	Age (years;months)	Instrument†	Type of study
Bangladesh^16^	1	1862	18	Bayley-II	5;3–5;8	WPPSI			Program evaluation
Brazil 1^7^	1‡	644	3	Denver-II			18;6	WAIS	Birth cohort
	2	1412	6	Denver-II					
	3	1362	12	Denver-II					
Brazil 2^8^	1‡	3907	12	BDI-2					Birth cohort
	2‡	3869	24	BDI-2					
Chile 1^17^	1	128	6	Bayley-I	5;6–5;8	WPPSI			Program evaluation
	2	1732	12	Bayley-I					
	3	279	18	Bayley-I					
Chile 2^9^	1‡	4869	7–23	BDI-2					Birth cohort
	1§	9201	24–58	Tepsi	4;1–6;2	TVIP			
China^18^	1	990	18	Bayley-III					Program evaluation
Colombia 1^19 42^	1	704	12–24	Bayley-III	4;6–5;8	TVIP			Program evaluation
	2	631	24–42	Bayley-III					
Colombia 2^15^	1	1311	6–42	Bayley-III	6;0–8;11	WISC-V, TVIP			Instrument validation
	1	658	6–42	Denver-II					
	1	658	6–42	ASQ-3					
	1‡	635	6–42	BDI-2 Screener					
Ecuador^20^	1	667	0–35	Barrera	5;7–8;8	TVIP	9;2–12;2	TVIP	Program evaluation
Ethiopia^10 11^	1	193	12	Bayley-III			9;10–10;10	PPVT	Birth cohort
	2	440	30	Bayley-III					
	3	456	42	Bayley-III					
Jamaica 1^21^	1	225	15	Griffiths	6;6–7;1	WPPSI, PPVT			Program evaluation
	2	218	24	Griffiths					
Jamaica 2^22^	1	159	9–24	Griffiths	7;0–8;3	SB-4, Raven’s, PPVT	16;9–18;0	WAIS	Program evaluation
	2	159	21–36	Griffiths					
	3	159	33–48	Griffiths					
Madagascar^23^	1	205	34–42	SB-5	6;10–7;10	SB-5, PPVT			Program evaluation
Netherlands 1^14^	1	1985	1	DDI	4;7–6;1	UKKI			Instrument validation
	2	1807	2	DDI					
	3	1963	3	DDI					
	4	1919	6	DDI					
	5	1881	9	DDI					
	6	1802	12	DDI					
	7	1776	15	DDI					
	8	1787	18	DDI					
	9	1815	24	DDI					
Netherlands 2^12^	1	1016	24	DDI					Birth cohort
	2	995	30	DDI					
	3	1592	36	DDI					
	4	1534	42	DDI					
	5	1034	48	DDI					
South Africa^13^	1	485	6	Bayley-I, Griffiths	4;10–5;6 7;0–8;6	Denver-II Raven’s (Coloured)			Birth cohort
	2	275	12	Bayley-I, Griffiths					
	3	1802	24	Vineland					
	4‡§	1614	48	Vineland					

*Early childhood testing instruments were the ASQ-3; Barrera; BDI-2 and BDI-2 Screener; Bayley-I, II and III; Denver-II, DDI; Griffiths; SB-5; Tepsi; and the Vineland. References for instruments are included with online supplementary appendix table A1.

†Middle childhood and adolescence testing instruments were the Denver-II; PPVT and the Spanish version, TVIP; Raven’s and Raven’s (Coloured); SB-4 and SB-5; UKKI; WAIS; WISC-V; and WPPSI. References for instruments are included with online supplementary appendix table A1.

‡These rounds of data that were not used in the final 565-item D-score model.

§Excludes children>48 months at Time 1 from validity tests.

ASQ-3, Ages & Stages Questionnaires; Barrera, Barrera Moncada; Bayley-I, II and III, Bayley Scales for Infant and Toddler Development; BDI-2, Battelle Development Inventory and Screener-2; DDI, Van Wiechenschema, referred to as the Dutch Developmental Instrument; Denver-II, Denver Developmental Screening Test; D-Score, Developmental Score; Griffiths, Griffiths Mental Development Scales; PPVT, Peabody Picture Vocabulary Test; Raven's, Raven's Progressive Matrices; SB-4 and SB-5, Stanford Binet Intelligence Scales; Tepsi, Test de Desarrollo Psicomotor; TVIP, Test de Vocabulario en Imagenes Peabody; UKKI, Utrechtse Korte Kleuter Intelligentietest; Vineland, Vineland Social Maturity Scale; WAIS, Wechsler Adult Intelligence Scale; WISC-V, Wechsler Intelligence Scale for Children - Revised; WPPSI, Wechsler Preschool and Primary Scale of Intelligence.

10.1136/bmjgh-2019-001724.supp1 Supplementary data

Study cohorts were purposively selected for inclusion in this study if children were assessed with a direct assessment instrument at least once during early childhood (<48 months, Time 1) and again during middle childhood to adolescence (Time 2) when they were ages >4–18 years. Availability of item-level assessment data at Time 1 was also a requirement. We included item-level data for children ≥36 months at Time 1 to ensure items were included that high-performing 3-year-old children would fail. In some cohorts, multiple instruments were used and/or multiple rounds of data were collected at Time 1 (eg, at 6, 12, 18, and 24 months) and Time 2. All data from Time 1 were included in the D-score model building process (see below). Available data for children 48–58 months at Time 1 were excluded from validity tests as our aim was to create a metric for young children that would be predictive of their skills at ages over 4 years.

### Item instrument mapping

Instruments used in each study were internationally recognised and locally adapted for assessing development of young children using multiple items (table 1 and online supplementary appendix table A1 with associated references). Instruments were primarily direct assessment, with two caregiver-report instruments (Ages and Stages Questionnaire or ASQ and Vineland Social Maturity Scale). A list of instruments and corresponding citations are provided in the supplementary materials. Although published separately, these instruments incorporate many similar items designed to assess the same developmental skills, a critical feature required for linking across disparate datasets.

Advisory Board members created a master spreadsheet of >1500 items administered with instruments at Time 1, organised across five developmental domains: fine motor, gross motor, receptive language, expressive language, and cognition. Personal-social development was not included, as measures of this domain were inconsistently used across the cohorts. Although early personal-social development facilitates rich child-caregiver interactions, the expression and interpretation of personal-social development vary across cultures.^24^

Within each of the five domains, individual items from each instrument (eg, Denver Developmental Screening Test or Griffiths Mental Development Scales) were mapped to same-skill items in the Bayley Scales of Infant and Toddler Development, third edition (Bayley-III), which was the most frequently administered instrument. Equivalency of skills between items was determined by referring to manuals, item descriptions and extensive hands-on testing experience by Board members. We also mapped groups of same-skill items across other instruments that did not map onto Bayley-III items. Caregiver-report items were mapped to direct assessment items if the skill assessed was considered equivalent. The mapping exercise resulted in 95 groups of items from different scales measuring the same skill termed ‘equate groups’, each containing at least two same-skill items from different instruments (eg, item ‘stacks 2 cubes’ in Instrument A=item ‘builds 2 block tower’ in Instrument B).

### Data harmonisation

The master spreadsheet of Time 1 items formed the basis for combining the data from the 16 cohorts into a single database, with equate groups identified to link items across instruments and cohorts. All items were coded as 0 (fail), 1 (pass) or missing. In the Battelle Developmental Inventory, items were originally scored as 0 (fail) with passing scores of 1 or 2 depending on the level of skill demonstrated or time taken to complete the task. For all Battelle items, 2 was recoded as 1. For six Battelle items, a score of 1 was recoded as 0 because these items were mapped to Bayley-III items that were more difficult. Similarly, ASQ items were originally scored as 0 (not yet), 5 (sometimes) and 10 (succeeds); both 5 and 10 were recoded as 1. Harmonisation resulted in a matrix with 71 403 rows (child-round observations) and 1572 columns (items) collected from 36 345 unique children. Since each cohort and round of data collection yielded information on a subset of items, by design, the matrix included many empty cells.

### Model building and active equate groups

A unidimensional statistical model for the D-score was built using the Rasch model, a simple logistic model for which an observed response is a function of the difference between person ability and item difficulty.^25^ In the Rasch model, when a person’s ability is equal to the item difficulty, there is a 50–50 chance of passing that item. Probability of passing is above 50% when ability is greater than the item difficulty and below 50% when ability is lower than the item difficulty. To convert scores from different instruments to a common scale, we applied psychometric equating methods typically used in educational testing.^26^ Instrument equating in our application required the identification of equate groups with comparable psychometric performance for all items in the group, across instruments and cohort origins, such that group items could be statistically constrained to have the same difficulty.^27^ We defined these equate groups as ‘active’ as they mathematically bridge instruments and cohorts, linking them to a common scale. Children with the same underlying developmental ability should have the same probability of passing active equate items.

Model building was a multistep process. We removed 233 items with fewer than 10 observations in the least populated response category (ie, pass or fail), leaving 1339 items. Next, we evaluated progressively refined Rasch models that varied along two dimensions: (1) the subset of active equate groups and (2) the cut-points for item fit statistics (ie, residual (outfit) and weighted (infit) mean square fit) used to exclude poorly fitting items from the model. To optimise measurement properties, we limited activation of equate groups to those that performed very well across instruments and cohorts, rather than activating ones with variable performance. Also, we sought active equate groups representative of the five developmental domains of interest and of abilities of children across the age range 0–47 months. Finally, we aimed for active equate groups to connect instruments by at least three items.

In the final Rasch model, items were retained if they were part of an active equate group or included as independent items if both their infit and outfit statistic were <1. Items from equate groups that were not activated (ie, passive equate groups) were not constrained to the same difficulty and treated as independent items. Independent items from a single instrument administered in more than one country were statistically constrained to a single difficulty (eg, Bayley-III items administered in China, Colombia and Ethiopia) if children with the same latent ability (but not necessarily of the same age) were found to have the same probability of passing these items regardless of country of origin. By constraining the difficulty of same-instrument items in the model, we gain additional links to the common scale for cohorts from different countries who were administered the same instrument. Independent items also improve the precision of estimated D-scores. Items with poor fit to the Rasch model or demonstrating differential performance by country were excluded from the final model and analyses of validity.

### D-score and DAZ estimates

For each child at each round, a D-score was estimated from the final model by the expected a posteriori (EAP) method.^28^ To establish the numerical range for the scale, we anchored the D-score relative to two indicators that are used widely in different instruments and are easy to measure and minimally sensitive to cultural variation: ‘lifts head to 45 degrees in prone position’ and ‘sits in stable position without support’. Fixed item difficulties of 20 and 40 D-score units were used for these items, respectively, based on previous analyses of the Netherlands 1 cohort data.^29^ These values were chosen such that D-scores start near zero at age 1 month. In the first year of life, a one D-score unit increase corresponds to approximately 1 week difference in age. In the second year of life, a one unit increase corresponds to approximately 1 month. Regardless of age, a 10-unit increase in the D-score corresponds to a change from children being very likely to fail (>90%) an item to very likely to pass (>90%).

We modelled the age-conditional distribution of D-scores across country cohorts with the Lambda-Mu-Sigma (LMS) method,^30^ an accepted approach for fitting growth curves, to generate a D-score-for-age z-score (DAZ) for each child.

### Validation

DAZ estimates for children aged <48 months at Time 1 were used to examine discriminant, concurrent and predictive validity of the D-score metric. There were 35 rounds of data collection across the 16 cohorts (referred to henceforth as data rounds). Discriminant validity was examined by comparing mean DAZ by three predictors of early child development:^31^ low birth weight (<2.5 kg), stunting (height-for-age<−2 SD of median WHO Growth Standards for same age and sex children)^32^ and maternal education (no education, any primary, any secondary and above secondary education). The maternal education classification chosen could be consistently applied across the available studies, with categories in some cohorts having small samples. Household wealth was captured in all studies, but wealth was estimated in ways that were not comparable across settings. Other predictors of early development, such as gestational age and nurturing care indicators,^33^ were considered but were not generally available across studies. We used t-tests for low birth weight and stunting, and analysis of variance F-tests for maternal education, to evaluate whether DAZ was sensitive to differences in child ability across these established risk/protective factors for early childhood development,^31^ with significance set at p<0.05. Scores for categories with fewer than 10 observations were excluded from tests of significance.

For concurrent validity, we calculated pairwise Pearson correlations of DAZ with age-standardised scores for the original instruments. When available, we used standardised scores based on external standards. Otherwise, we generated age-adjusted z-scores for a given cohort (internal standardisation) using non-parametric methods.^15^ For the Netherlands 1 cohort, the 9 rounds of data collection were collapsed into three 12-month age intervals, which did not change results.

For predictive validity, we correlated DAZ by data collection round at Time 1 with standardised test scores acquired at Time 2 in middle childhood (>4–9 years) and adolescence (>9–18 years). Time 2 data were included in prediction analyses if ≥2 years had passed since Time 1 data collection. Because initial age of testing affects prediction of later outcomes,^34 35^ cross-sectional data covering a wide age range at Time 1 in Chile 2, Colombia 2 and Ecuador were grouped into 12-month age intervals. The data collection rounds of the Netherlands 1 cohort were collapsed as explained above. Although originally planned for the analysis, China Time 2 data were not ready to be shared for this project. Time 2 assessments (see table 1) include tests of IQ (eg, Wechsler Preschool and Primary Scale of Intelligence), matrix reasoning (Raven’s Coloured Progressive Matrices) and receptive vocabulary (Peabody Picture Vocabulary Test).

For both concurrent and predictive validity, we classified correlations as low (*r*=0.20–0.39), moderate (*r*=0.40–0.59), strong (*r*=0.60–0.79) or very strong (*r*=0.80–1).^36^

### Software

All model fitting and evaluation of items and equate groups was done with R. We extended the function sirt::rasch.pairwise.itemcluster^37^ with an option to constrain the solution by equate groups. See Eekhout, Weber and van Buuren (under review)^27^ for more details. Tests of validation were performed in R or Stata V 14.

### Role of the funding source

The Bill and Melinda Gates Foundation (BMGF) approved the study design as part of funding approval, but had no role in data collection, analysis, interpretation or write-up of results or in the decision to submit the paper for publication.

### Ethical considerations

The study involved secondary data analyses of deidentified data. Investigators signed a data sharing agreement stating that they had approval to use these data for this project from study collaborators and/or institutions. Approval for the secondary analyses was obtained from the Netherlands Organization for Applied Research (TNO) and the ethical review board at Stanford University.

### Patient and public involvement

This non-clinical research was performed using deidentified data from completed studies without patient or public involvement. No new participants were recruited and no new data were collected.

## Results

The final model used to estimate D-scores contained 565 items originating from 11 instruments and included 18 active equate groups. The number of items administered for any given child varied considerably across and within cohorts, with an overall average of 27 items per child used to estimate their D-score (country per child averages ranged from 3.5 items in Ecuador to 59.5 items in Bogota where multiple instruments were used, see online supplementary Table A2). Items from the Battelle Developmental Inventory performed poorly in the model and were removed from further analysis, resulting in the loss of one cohort (Brazil 2) as well as Battelle data from Colombia 2 and Chile 2.

**Figure 1:**
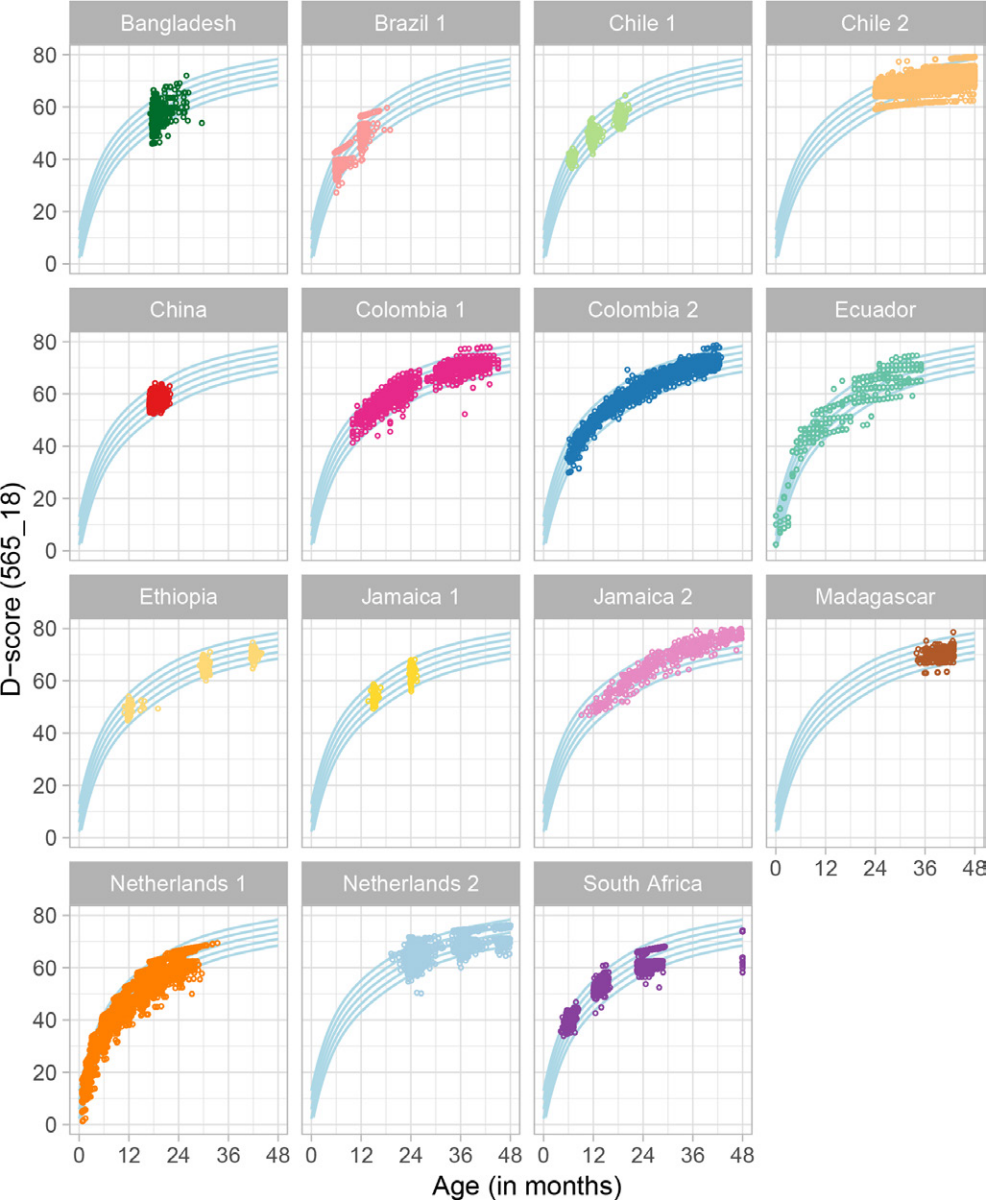
Distribution of the D-score by age and cohort with the final model (565 items and 18 equate groups). D-score, Developmental Score.

The plots in figure 1 show the distribution of D-score estimates by age and cohort applying the final model to all rounds of Time 1 data. The blue curved lines represent the age-conditional distribution of the combined dataset for all cohorts and are driven by the large Colombia 2 and Chile 2 samples. Average D-score trajectories from the Netherlands 1 and Colombia cohorts follow the age-conditional distribution of the combined dataset across a ≥2 year age range. Distributions of scores in the other cohorts reflect study sampling and data availability, but generally fall within the age-specific percentiles developed for the full dataset. For example, the China cohort was assessed at 18 months at Time 1 such that all D-scores are grouped together around that age. In contrast, the Ethiopia cohort was assessed at 12, 30 and 42 months and the plot shows three groupings of scores that increase on average with age.

### Discriminant validity

The overall mean DAZ for all cohorts combined is 0 and the range is from −7 to +4.5 SD units. The mean DAZ and SDs by birth weight, stunting and maternal education are shown in table 2 for cohort rounds with available data. Children above the low birth weight cut-point demonstrated significantly higher mean DAZ scores than those below the cut-point in 18 of 26 data rounds (69%) with available data. Non-stunted children had significantly higher scores than stunted children in 21 of 28 (75%) data rounds. DAZ scores increased significantly with maternal education in 17 of 31 (55%) data rounds. In another six data rounds, mean DAZ increased with maternal education, but differences were not statistically significant.

**Table 2:** Discriminant validity of DAZ with birth weight, nutritional status and maternal education

Birth weight†					Nutritional status†			Maternal education				
Country	Round	Low	Normal	P value	Stunted	Non-stunted	P value	No education	Any primary	Any secondary	Above secondary	P value
Bangladesh	1	−**0.71** (0.04) 510	−**0.51** (0.03) 1317	<0.001	−**0.73** (0.03) 820	−**0.42** (0.03) 1007	<0.001	−**0.73** (0.04) 625	−**0.67** (0.05) 397	−**0.41** (0.04) 783	**0.75** (0.25) 22	<0.001
Brazil	2	**0.23** (0.86) 415	**0.73** (0.79) 994	<0.001	**0.09** (0.9) 218	**0.67** (0.8) 1194	<0.001	**0.26** (0.77) 35	**0.55** (0.85) 1031	**0.63** (0.81) 242	*	0.041
	3	**0.37** (0.88) 401	**0.87** (0.87) 959	<0.001	**0.18** (1.01) 184	**0.81** (0.85) 1177	<0.001	**0.2** (0.91) 32	0.69 (0.87) 988	0.79 (1) 240	*	0.002
Chile 1	1	*	0.51 (0.46) 128	N/A	*	0.5 (0.47) 126	N/A	*	0.57 (0.4) 48	0.48 (0.51) 69	0.42 (0.42) 10	0.511
	2	*	0.16 (0.53) 1611	N/A	0.01 (0.46) 12	0.16 (0.53) 1409	0.331	*	0.14 (0.49) 601	**0.16** (0.55) 838	**0.26** (0.52) 163	0.037
	3	*	−0.27 (0.7) 278	N/A	*	−0.27 (0.7) 225	N/A	*	−0.36 (0.68) 109	−0.21 (0.72) 147	−0.15 (0.65) 21	0.212
Chile 2	1	−0.12 (0.99) 327	−0.02 (0.91) 8071	0.077	−0.14 (0.99) 292	−0.03 (0.91) 8383	0.068	−**0.01** (0.99) 100	−**0.35** (0.92) 1717	−**0.05** (0.89) 5245	**0.24** (0.90) 2139	<0.001
China	1	0.06 (0.56) 9	−0.08 (0.58) 968	0.464	−0.2 (0.47) 29	−0.08 (0.59) 943	0.253	*	−**0.31** (0.57) 34	−**0.1** (0.56) 742	**0.06** (0.64) 178	<0.001
Colombia 1	1	**−0.11** (0.78) 47	**0.29** (0.96) 593	0.001	**0.001** (1.07) 99	**0.28** (0.92) 591	0.015	−**0.23** (0.92) 27	**0.1** (0.96) 238	**0.33** (0.93) 375	*	<0.001
	2	0.09 (0.93) 43	0.2 (0.93) 535	0.495	−**0.18** (1.31) 59	**0.21** (0.86) 569	0.032	−**0.29** (1.01) 21	−**0.06** (0.93) 217	**0.29** (0.87) 337	*	<0.001
Colombia 2	1	−**0.1** (0.77) 128	**0.14** (0.78) 1062	0.001	−**0.03** (0.78) 229	**0.13** (0.8) 1080	0.005	*	−**0.23** (0.82) 140	**0.06** (0.78) 744	**0.32** (0.78) 393	<0.001
Ecuador	1	0.5 (1.05) 12	0.38 (1.48) 133	0.733	−**0.17** (1.45) 89	**0.44** (1.49) 293	0.001	−**0.27** (2.04) 14	**0.19** (1.45) 433	**0.65** (1.32) 197	**0.70** (1.56) 11	<0.001
Ethiopia	1	−0.26 (0.45) 16	−0.08 (0.6) 174	0.152	−0.13 (0.64) 84	−0.08 (0.56) 103	0.583	−0.07 (0.6) 168	−0.29 (0.5) 22	*	*	0.102
	2	−0.59 (0.78) 33	−0.34 (0.56) 407	0.088	−**0.4** (0.58) 328	−**0.27** (0.59) 111	0.042	−**0.39** (0.6) 385	−**0.19** (0.46) 54	*	*	0.017
	3	−**0.8** (0.5) 35	−**0.58** (0.46) 421	0.017	−**0.65** (0.47) 345	−**0.44** (0.42) 109	<0.001	−**0.62** (0.46) 402	−**0.45** (0.47) 53	*	*	0.016
Jamaica 1	1	**0.04** (0.53) 131	**0.22** (0.49) 94	0.01	−**0.17** (0.45) 14	**0.14** (0.52) 210	0.026	*	*	0.12 (0.5) 216	*	N/A
	2	**0.35** (0.78) 130	**0.55** (0.68) 88	0.044	*	0.44 (0.74) 215	N/A	*	*	0.46 (0.71) 208	*	N/A
Jamaica 2	1	ND	ND		−**0.23** (0.76) 122	**0.55** (0.57) 37	<0.001	*	−0.03 (0.77) 138	−0.15 (0.88) 21	*	0.54
	2	ND	ND		0.78 (1.02) 62	0.97 (0.87) 97	0.213	*	0.93 (0.96) 138	0.73 (0.75) 21	*	0.36
	3	ND	ND		1.61 (0.99) 48	1.86 (0.98) 111	0.141	*	1.81 (0.98) 138	1.66 (1.08) 21	*	0.52
Madagascar	1	ND	ND		−0.39 (0.94) 113	−0.23 (0.9) 89	0.205	−0.53 (0.84) 51	−0.35 (0.99) 118	−0.03 (0.89) 35	*	0.054
Netherlands 1	1	−**1.02** (1.02) 97	−**0.23** (1.02) 1888	<0.001	−**0.87** (1.04) 96	−**0.17** (1) 1264	<0.001	*	−0.22 (1.01) 602	−0.28 (1.05) 1008	−0.32 (1.06) 328	0.335
	2	−**0.48** (1.18) 69	**0.06** (0.9) 1689	<0.001	−**0.42** (1.32) 75	**0.07** (0.92) 1149	0.003	*	0.02 (0.94) 520	0.08 (0.88) 901	−0.03 (0.99) 299	0.157
	3	−**0.83** (1.1) 106	−**0.03** (0.9) 1851	<0.001	−**0.46** (1.07) 120	−**0.05** (0.9) 1793	<0.001	*	−0.09 (0.94) 593	−0.1 (0.92) 994	0.02 (0.94) 325	0.156
	4	−**0.59** (1.08) 105	−**0.05** (0.92) 1809	<0.001	−**0.41** (0.93) 49	−**0.07** (0.93) 1830	0.011	*	−**0.15** (0.97) 579	−0.09 (0.94) 973	**0.1** (0.84) 320	<0.001
	5	−**1.01** (1.1) 110	−**0.25** (0.95) 1766	<0.001	−**0.83** (1.33) 43	−**0.28** (0.96) 1804	0.011	*	−**0.3** (1.03) 566	−**0.34** (0.97) 965	−**0.14** (0.91) 306	0.006
	6	−**0.92** (1.11) 98	−**0.37** (1.07) 1694	<0.001	−**0.99** (1.5) 30	−**0.39** (1.06) 1739	0.002	*	−0.43 (1.12) 540	−0.42 (1.06) 924	−0.28 (1.03) 292	0.104
	7	−**0.77** (1.14) 97	−**0.37** (1.06) 1669	0.001	−**1** (1.7) 33	−**0.38** (1.05) 1706	0.001	*	−0.47 (1.11) 528	−0.39 (1.04) 918	−0.3 (1.06) 286	0.079
	8	−**0.68** (1.07) 95	−**0.22** (1.1) 1580	<0.001	*	−0.4 (1.09) 40	N/A	*	−**0.36** (1.09) 508	−**0.24** (1.11) 871	−**0.05** (1.1) 269	<0.001
	9	−0.25 (1.25) 106	−0.02 (1.12) 1703	0.059	−**1.15** (1.84) 16	−**0.01** (1.12) 1746	0.026	*	−**0.3** (1.16) 557	**0.04** (1.1) 925	**0.32** (1.04) 293	<0.001
South Africa	1	**0.69** (0.55) 51	**1.01** (0.64) 433	<0.001	ND	ND		*	**0.98** (0.74) 55	**1.01** (0.62) 376	**0.71** (0.6) 41	0.020
	2	0.48 (0.82) 17	0.5 (0.7) 257	0.946	**0.06** (1.02) 12	**0.56** (0.71) 137	0.026	*	0.33 (0.59) 33	0.49 (0.74) 206	0.71 (0.52) 32	0.096
	3	−**0.24** (1.04) 188	−**0.02** (1.03) 1609	0.007	−**0.11** (1.06) 299	**0.08** (1.02) 1020	0.006	−0.02 (1.01) 20	−0.13 (1.11) 224	−0.05 (1.02) 1337	0.08 (1.01) 215	0.229

*<10 observations, N/A=Not applicable, ND=No data available. Differences significant <0.05 are in bold.

†Low birth weight is defined as <2.5 kg and stunted is height-for-age z-score of <−2 SD of the median WHO Growth Standards for same-age and same-sex children.^32^

Subgroup data are mean (SD) n.

### Concurrent validity

Moderate to strong concurrent validity was anticipated as the D-score is computed from subsets of items from original instruments (table 3). The proportion of items from the original instrument used to estimate the D-score for each cohort averaged 0.61 and ranged from 0.13 to 1.0. The average concurrent validity of the DAZ with standardised scores from the original instruments was strong (r=0.71), ranging from 0.24 to 0.96. Results were robust to the use of externally and internally standardised scores in the Colombia 1 and 2 cohorts, which allowed for both methods of standardisation (not shown).

**Table 3:** Concurrent correlation of DAZ in children under 48 months with measures from original instruments

Cohort	Age range (months)	% items in D-score from original instrument*	Bayley-I, II, III†			Other measures	
			Cognition	Language	Motor		
			MDI		PDI	Total Score	Measure
Bangladesh	18	0.13 (16/121)	0.797		0.503		
Brazil 1	5–11	1 (18/18)				0.859	Denver-II
	11–19	0.84 (16/19)				0.926	
Chile 1	6	0.67 (31/46)	0.861		0.438		
	12	0.55 (60/109)	0.880		0.361		
	18	0.45 (26/58)	0.835		0.249		
Chile 2‡	24–35	0.53 (33/62)				0.768	Tepsi
	36–47					0.855	
China	18	0.53 (40/76)	0.541	–	0.458		
Colombia 1	10–26	0.41 (104/254)	0.710	0.809	0.775		
	28–45	0.45 (96/212)	0.742	0.840	0.672		
Colombia 2	6–17	0.32 (200/631)	0.386	0.333	0.675	0.758	Denver-II
	18–29		0.671	0.837	0.651	0.642	
	30–42		0.649	0.811	0.620	0.795	
Ecuador	0–11	0.68 (15/22)				0.791	Barrera
	12–23					0.815	
	24–35					0.768	
Ethiopia	11–12	0.48 (73/151)	0.614	0.560	0.915		
	29–32	0.46 (83/181)	0.737	0.814	0.808		
	41–44	0.42 (61/146)	0.631	0.723	0.696		
Jamaica 1	15	0.47 (69/148)				0.930	Griffiths DQ§
	24	0.44 (68/155)				0.862	
Jamaica 2	9–25	0.44 (94/212)				0.574	Griffiths DQ§
	21–37	0.33 (66/200)				0.888	
	33-48	0.29 (52/181)				0.864	
Madagascar	34–42	0.24 (10/41)				0.452	SB-5
Netherlands 1¶	0–11	0.57 (4/7) to 1 (13/13)				0.949	DDI
	12–23	0.92 (12/13)				0.958	
	24–34	0.71 (10/14)				0.486	
South Africa	6	0.48 (80/166)	0.791		0.775	0.868	Griffiths DQ§
	12	0.5 (101/202)	0.763		0.659	0.725	
	24	0.41 (12/29)				0.729	Vineland

*Not all items from the original instrument were included in the final model. Items from multiple instruments were included in D-score estimation in Colombia 2 and South Africa.

†Bayley-I, II, III scores are externally standardised, except for Ethiopia, which is internally age-standardised. Externally standardised scores are cognitive, language and motor composite scores for the Bayley-III; and the MDI and PDI for the Bayley-I and Bayley-II.

‡Excludes children>48 months at Time 1.

§Griffiths DQ includes motor development items.

¶Data collection rounds for Netherlands 1 were collapsed into 1 year age increments (0–11 m, 12–23 m, 24–34 m). Range of % items from original instrument used in D-score varies by round, but concurrent correlation is ≥0.9 for all collapsed rounds. 32 months was the maximum age in completed months for the test of predictive validity.

Barrera, Barrera Moncada; Bayley-I, II, III, Bayley Scales for Infant and Toddler Development; DDI, Van Wiechenschema, referred to as the Dutch Developmental Instrument; Denver-II, Denver Developmental Screening Test; DQ, Developmental Quotient; D-Score, Developmental Score; Griffiths, Griffiths Mental Development Scales; MDI, Mental Development Index; PDI, Psychomotor Development Index; SB-5, Stanford Binet Intelligence Scales; Tepsi, Test de Desarrollo Psicomotor; Vineland, Vineland Social Maturity Scale.

### Predictive validity

**Figure 2:**
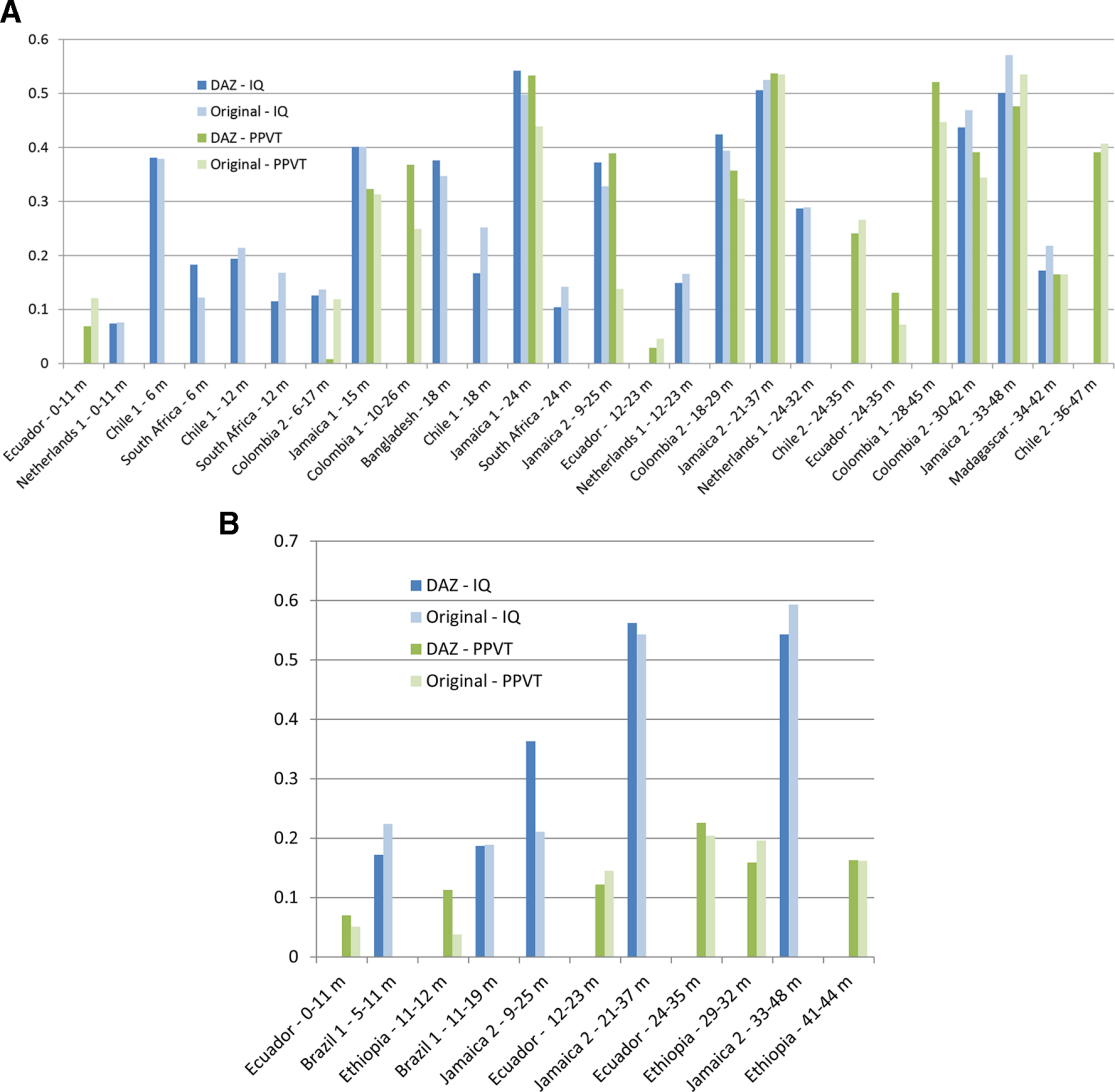
(A) Correlations of DAZ and age-adjusted original measures of early childhood development in children under 48 months with IQ and receptive vocabulary measures at Time 2 for ages >4–9 years, arranged by age at Time 1. For the original instruments, Bayley-I and Bayley-II, we used the MDI in the correlations (Bangladesh, Chile 1 and South Africa); for Bayley-III, we used the measure from the cognition domain (Colombia 1 and Colombia 2). (B) Correlations of DAZ and age-adjusted original measures of early childhood development in children under 48 months with IQ and receptive vocabulary measures at Time 2 for ages >9–18 years, arranged by age at Time 1. For the original instrument, Bayley-III, we used the measure from the cognition domain (Ethiopia). IQ measures are Denver-II, Raven’s and Raven’s (Coloured), SB-4 and SB-5, UKKI, WAIS, WISC-V, and WPPSI. Receptive language measures are the PPVT and its Spanish version, TVIP. Bayley-I, II and III, Bayley Scales for Infant and Toddler Development; Denver-II, DenverDevelopmental Screening Test; DAZ, D-score-for-age z-scores; D-Score, Developmental Score; IQ, Intelligence Quotient; MDI, Mental Development Index; PDI, Psychomotor Development Index; PPVT, Peabody Picture Vocabulary Test; Raven’s, Raven’s Progressive Matrices; SB-4 and SB-5, Stanford Binet IntelligenceScales; TVIP, Test de Vocabulario en Imagenes Peabody; UKKI, Utrechtse Korte Kleuter Intelligentietest; WAIS, Wechsler Adult Intelligence Scale - Revised; WISC-V, Wechsler Intelligence Scale for Children; WPPSI, Wechsler Preschool and Primary Scale of Intelligence

The figure 2A and B presents predictive validity to measures of IQ and receptive vocabulary in middle childhood (Time 2 for ages >4–9 years) and adolescence (Time 2 for ages >9–18 years), respectively, of both DAZ and the original instruments. When multiple scores were available for original instruments, we included the cognitive score (eg, over language or motor) or the Bayley-III score (eg, over the Denver-II in Colombia 2). Detailed tables are included in the online supplementary appendix, table A3a for DAZ and online supplementary appendix, table A3b for scores obtained from original instruments.

The average predictive correlation of the DAZ with IQ and receptive vocabulary scores in middle childhood was 0.29 (range 0.07–0.54) and 0.31 (range 0.008–0.54), respectively. Predicting to adolescence, the average correlation of the DAZ with IQ and receptive vocabulary was 0.37 (range 0.17–0.56) and 0.14 (range 0.07–0.23), respectively. The DAZ performed, as well as occasionally outperformed, single dimension scores from the original instruments. For example, in Colombia 1, the correlation of the 10–26 month DAZ with later receptive language (0.368) was comparable or slightly larger than correlations of age-standardised cognition, language and motor subscale scores from the Bayley-III with the later measure (0.277, 0.322 and 0.278, respectively). In Brazil 1, correlations of age-standardised cognitive and language subscale scores from the Denver-II with IQ at age 18 years were only 0.051 and 0.127, whereas correlations for the DAZ and the composite measure from the Denver-II were similar (0.187 and 0.189). In general, the correlation of the DAZ with Time 2 measures increased with age at Time 1 within a given cohort. However, the age trend was not consistent across cohorts.

### Simulation of a new instrument

Although the number of items administered to each child varied considerably between cohorts, children’s D-score was estimated from an average of 55% fewer items than the average 55 items used in the original instruments. For example, in the Jamaica 1 cohort, information was available for, on average, 87 items per child, and yet the D-score was calculated, on average, from 43 items per child. The ability to reduce the items needed to estimate the D-score suggests the feasibility of creating a relatively short instrument for future field work. We simulated this by obtaining estimated D-scores on a subset of items included in the final D-score model. First, final model items were sorted by age equivalence (ages at which 10% pass, 50% pass and 90% of children pass each item) and reviewed by Advisory Board members to retain items that were non-duplicative of a skill, easy to train and administer, feasible for use in the field, and likely to demonstrate cross-cultural validity. The subset of 165 items comprised approximately 20–25 items per 6-month age group. The simulation showed that D-score estimates from this subset were very strongly correlated (r=0.999) with the full 565-item model.

## Discussion

The development of the D-score was driven by the need for a valid and easily interpretable metric for an underlying latent construct of infant and toddler development that is comparable across cultures and contexts. A statistical model for the D-score was constructed that mathematically bridges data from multiple internationally recognised and commonly used instruments, using a set of linking items that performed equivalently across countries and cohorts. By leveraging existing longitudinal data for >36 000 children from 11 low-income, middle-income and high-income countries, we produced a common metric of early childhood development with acceptable discriminant and concurrent validity. Children from diverse countries were shown to have similar developmental profiles with increasing age, supporting theories of a universal unfolding of stage-based skills in the first few years of life that is responsive to environmental and cultural variation.

A primary strength of this study was the use of existing longitudinal data from early childhood (<4 years) and again during middle childhood and adolescence (>4–18 years), circumventing the high cost and time associated with obtaining new data prospectively. Critically, the interval-scale property of the D-score enables quantitative comparisons across ages, which in turn will allow for the construction of international standards for children’s healthy development in the future. Using the D-score, depictions of children’s developmental trajectories with age are easy to interpret, unlike scores obtained from conventional instruments that employ age-based standardisation.

In further contrast to conventional instruments, which are typically designed and validated in a single country or region, data for this study encompassed cohorts from multiple countries and contexts, reflecting children’s development across a diverse global sample. Although representation from high income countries was limited to one country, an innovative feature of the statistical model is that it enables the estimation of D-scores for other item-level datasets not included in this project. Such use of the model will enable external validation in new contexts. A user-friendly open-source platform and algorithm that allows users to generate D-scores from item-level data obtained in their sites is under development (preliminary access to the algorithm is available at https://github.com/stefvanbuuren/dscore).

Although a strength, the use of existing data also represents one of the study’s limitations: validation results were affected by differences in sampling strategies across studies (eg, inclusion criteria for low-income and low-middle-income families in Bogota or selection based on children’s stunting status in Jamaica). Nonetheless, predictive validity of the D-score metric to later IQ and language outcomes was comparable to that obtained with the original instruments from which the metric was derived. It improved with increasing age at Time 1, consistent with other reports, including those using the Bayley.^34 35^ Unexpectedly high correlations of 6-month age group children in the Chile 1 cohort may be a function of the study sampling children with and without iron-deficient anaemia, thus widening the distribution of scores across the whole sample. Similarly, high correlations in the Brazil 1 and Jamaica cohorts, even to 18 years, may be related to sampling groups of normal and low birth weight (Brazil 1 and Jamaica 1) and stunted and non-stunted (Jamaica 2) children.

Predictive correlations were low in some samples. In rural Africa, these may be explained either by low variability in the samples or from an education bias resulting in poor performance of the school-age instruments. In addition, cohorts in Ethiopia and Madagascar were assessed at Time 2 with adaptations of a receptive vocabulary test that is subject to item bias in countries with multiple languages or dialects.^38^ The low predictive correlation in Ecuador may be a function of measurement error due to the small number of items used in estimating the D-score in that cohort (as few as four items per child). Finally, the Dutch instrument was designed to screen children for developmental delay such that the high end of the D-score distribution was less well-represented than the low end.

We speculate that the poor performance of the Battelle in the model was due to its original 3-level item scoring, which made it difficult to map Battelle items precisely to items from the other instruments scored as pass/fail. Although some recoded Battelle items had reasonable fit to the Rasch model, in general, they did not equate well with other instruments or demonstrated differential performance by country.

The D-score metric and model set the stage for constructing new instruments with test items that are likely to demonstrate global validity. As we demonstrated with the simulation exercise, fewer items may be necessary than those included in existing conventional instruments, some of which are challenging to adapt to local languages and contexts. Furthermore, by relying on the D-score model’s predicted probability for successfully completing each item, we have the opportunity to incorporate adaptive or tailored testing with instruments based on the D-score. Model-based adaptive testing tailors the test to the child’s ability level by administering items based on success (or failure) in passing previously administered items. This approach allows for the rapid assessment of a child’s development with, for example, 10 or fewer items, while maintaining validity of the metric. Items are selected from the larger pool of items and targeted to the child’s age and individual pattern of passing items (ie, children of the same age may be administered different items depending on ability).

The D-score is currently being used by the Global Scale of Early Development (GSED) project to construct two new instruments. The first is intended as a population-level instrument for large-scale surveys, such as the Demographic Health Surveys or UNICEF’s Multiple Indicator Cluster Surveys, and will trade off precision in favour of speed and administrative simplicity (ie, using few items and caregiver-report). The second instrument will be for evaluations of small and large-scale programmes and policies.^39^ The programme evaluation instrument will be longer for better precision and will incorporate both caregiver-report and direct assessment, which takes longer and requires more administrative expertise, but avoids reporting bias, particularly when evaluating parenting programmes.

Instruments based on the D-score, such as the GSED, will allow for the new data collection necessary to develop standards from healthy populations and track country progress towards global goals of early childhood development. Although tracking progress can inform programmes and policies, the history of test score mis-use^40^ and the possibility of invalid and unfair conclusions drawn from cross-national comparisons should be acknowledged. Future examination of D-score trajectories will be most useful in highlighting environmental variations within and across countries, particularly in relation to poverty, education, nurturing care, and nutrition.

## Conclusion

With the recognition that critical building blocks for adult health and well-being are established early in life,^1^ countries throughout the world are instituting policies and programmes to ensure that all children reach their developmental potential. However, evaluating progress has been hampered by the lack of a validated metric of early childhood development across cultures, especially for children 0–3 years living in low- and middle-income countries (LMICs).^41^

The D-score metric and model aim to overcome this obstacle in two important ways. First, the D-score model can be used to convert existing data collected from multiple instruments across multiple settings to a common metric of early child development, advancing external validity. Second, the D-score can inform the selection of a subset of items from the larger pool of validated items in the model for constructing culturally-neutral, simple, fast and low-cost instruments, as with the GSED project. The inclusion of instruments based on a common metric in global surveys can ultimately lead to the data collection necessary to establish global standards for early childhood development.
