# Supplementary material for: The D-score: a metric for interpreting the early development of infants and toddlers across global settings
Source: BMJ Glob Health. 2019 Nov 19;4(6):e001724. doi: 10.1136/bmjgh-2019-001724 (PMC6882553; doi:10.1136/bmjgh-2019-001724)
Supplement: Supplementary data [file bmjgh-2019-001724supp001.pdf]

**Appendix: The D-score: a metric for interpreting the early development of infants and toddlers across global settings**

**Table A1: Name, abbreviation and citations for instruments during early childhood and at school-age**

| **Country/cohort** | **Early Childhood Instruments** | **Abbreviation** |
|---|---|---|
| Colombia 2 | Ages & Stages Questionnaires, 3rd edition[1] | ASQ-3 |
| Brazil 2, Chile 2, Colombia 2 | Battelle Developmental Inventory and Screener, 2nd edition[2] | BDI-2<br>BDI-2 Screener |
| Bangladesh, Chile 1, China, Colombia 1 & 2, Ethiopia, South Africa | Bayley Scales for Infant and Toddler Development, 1st, 2nd, and 3rd editions[3,4,5] | Bayley-I, II, and III |
| Brazil 1, Colombia 2 | Denver Developmental Screening Test, 2nd edition[6,7] | Denver-II |
| Ecuador | Barrera Moncada[8] | Barrera |
| The Netherlands 1 & 2 | Van Wiechenschema[9] (Dutch Developmental Instrument) | DDI |
| Jamaica 1 & 2, South Africa | Griffiths Mental Development Scales[10] | Griffiths |
| Madagascar | Stanford Binet Intelligence Scales version, 5th edition [11] | SB-5 |
| Chile 2 | Test de Desarrollo Psicomotor[12] | Tepsi |
| South Africa | Vineland Social Maturity Scale[13] | Vineland |
| **Country/cohort** | **School-age Tests** | **Abbreviation** |
| South Africa | Denver Developmental Screening Test, 2nd edition[6,7] | Denver-II |
| Jamaica 2 and South Africa | Raven's Progressive Matrices[14] and Raven's Coloured Progressive Matrices[15] | Raven's |
| Ethiopia, Jamaica 1, Madagascar | Peabody Picture Vocabulary Test (PPVT-III[16] and PPVT-4[17]) | PPVT |
| Chile 2, Colombia 1 & 2, Ecuador | Test de Vocabulario en Imagenes Peabody[18] (Spanish version of the PPVT) | TVIP |
| Jamaica 2 and Madagascar | Stanford Binet Intelligence Scales, 4th edition[19] and 5th edition[11] | SB-4<br>SB-5 |
| Brazil 1, Jamaica 2 | Wechsler Adult Intelligence Scale[20] | WAIS |
| Colombia 2 | Wechsler Intelligence Scale for Children-Revised[21] | WISC-V |
| Bangladesh, Chile 1, Jamaica 1 | Wechsler Preschool & Primary Scale of Intelligence, 3rd and 4th editions[22,23] | WPPSI |
| Netherlands 1 | Utrechtse Korte Kleuter Intelligentietest[24] | UKKI |

**Citations for Instruments**

1 Squires J, Bricker D, Twombly E, Nickel R, Clifford J, Murphy K, et al. Ages & Stages English Questionnaires, Third Edition (ASQ-3): A Parent-Completed, Child-Monitoring System. Baltimore, MD: Paul H. Brookes Publishing Co; 2009.

2 Newborg J. Battelle Developmental Inventory-2nd Edition. Rolling Meadows, IL: Riverside

Publishing, 2005.

3 Bayley N. Bayley Scales of Infant Development. New York: Psychological Corp, 1969.

4 Bayley, N. The Bayley Scales of Infant Development-II. San Antonio, TX: Psychological Corporation, 1993.

5 Bayley N. Bayley Scales of Infant and Toddler Development–Third Edition: Technical manual. San Antonio, TX: Harcourt Assessment, 2006.

6 Frankenburg WK, Dodds J, Archer P, Shapiro H, Bresnick B. The Denver II: a major revision and restandardization of the Denver Developmental Screening Test. *Pediatrics* 1992; **89**: 91–7.

7 Frankenburg WK, Dodds J, Archer P, *et al.* The DENVER II Technical Manual. Denver, CO: Denver Developmental Materials, 1990.

8 Barrera Moncada, O. 1981. Crecimiento y desarrollo psicológico del niño venezolano. Ediciones psicopediátricas. Caracas.

9 Schlesinger-Was EA. Ontwikkelingsonderzoek van zuigelingen en kleuters op het consultatiebureau. 1981.

10 Griffiths R. The Abilities of Babies: A Study in Mental Measurement. University of London Press, 1967.

11 Roid GH. Stanford–Binet Intelligence Scales, Fifth Edition. WPS Psychological Tests, 2003.

12 Haeussler IM, Marchant T. Tepsi: test de desarrollo psicomotor 2-5 años. Eds. Universidad Católica de Chile, 1999.

13 Doll EA. The measurement of social competence: A manual for the Vineland Social Maturity Scale. 1953.

14 Raven JC, Court JH. Raven's progressive matrices and vocabulary scales. Oxford pyschologists Press, 1998.

15 Raven JC. Coloured Progressive Matrices: Sets A, Ab, B. Oxford Psychologists Press, 2003.

16 Lloyd M. Dunn, Leota M. Dunn. "Peabody Picture Vocabulary Test-Third Edition (PPVT-III)". Pearson Education.

17 Lloyd M. Dunn, Douglas M. Dunn. "Peabody Picture Vocabulary Test, Fourth Edition (PPVT™-4)". Pearson Education.

18 TVIP Dunn LM, Lugo DE, Padilla ER, Dunn LM. Test de Vocabulario en Imagenes Peabody (TVIP), Pearson Clinical, 1986.

19 SB-4 Hagen E, Stattler J, Stanford–Binet Intelligence Scales, Fourth Edition·Thorndike, 1986.

20 Wechsler D. Wechsler Adult Intelligence Scale®-Fourth Edition. Pearson Education, 2008.

21 Wechsler D. Wechsler Intelligence Scale for Children®-Fifth Edition. Pearson Education, 2014.

22 Wechsler D. Wechsler Preschool and Primary Scale of Intelligence™ - Third Edition. Harcourt Assessment, Inc., San Antonio, TX, 2002.

23 Wechsler D. Wechsler Preschool and Primary Scale of Intelligence™ - Fourth Edition. Pearson Education, 2012.

24 Baarda, D.B. 1978. UKKI: Utrechtse Korte Kleuter Intelligentietest. Lisse: Swets en Zeitlinger.

**Table A2: Comparison of the average number of items administered to children and the average number of items included in the final Rasch model by study and overall.**

| Country/cohort | Number of items administered | Number of items in final model | Difference | Ratio |
|---|---|---|---|---|
| Bangladesh | 63.2 | 14.2 | 49 | 22% |
| Brazil 1 | 19.5 | 17 | 2.5 | 87% |
| Chile 1 | 42.1 | 30.4 | 11.7 | 72% |
| Chile 2 | 61.9 | 33 | 28.9 | 53% |
| China | 42.8 | 28 | 14.8 | 65% |
| Colombia 1 | 91.5 | 46.5 | 45 | 51% |
| Columbia 2 | 123.9 | 59.5 | 64.4 | 48% |
| Ecuador | 6.1 | 3.5 | 2.6 | 57% |
| Ethiopia | 79.5 | 38.6 | 40.9 | 49% |
| Jamaica 1 | 86.6 | 42.5 | 44.1 | 49% |
| Jamaica 2 | 100.8 | 38.5 | 62.3 | 38% |
| Madagascar | 29.5 | 7.5 | 22 | 25% |
| Netherlands 1 | 11 | 8.6 | 2.4 | 78% |
| Netherlands 2 | 16.8 | 14.2 | 2.6 | 85% |
| South Africa | 53.6 | 23 | 30.6 | 43% |
| Average | 55.3 | 27 | 28.3 | 55% |
| Min | 6.1 | 3.5 | 2.4 | 22% |
| Max | 123.9 | 59.5 | 64.4 | 87% |

Correlations of the early round scores (Time 1) with follow-up rounds (Time 2) are shown in Table A3a for the DAZ and Table A3b for the original instruments (e.g., Bayley-III, Denver-II). Because time between Time 1 and Time 2 varied considerably for different cohorts, Tables A3a and A3b were split into prediction to middle childhood (>4 to 9 years) and adolescence (>9 to 18 years). When multiple original domain measures were available, we included the cognitive measure (e.g., over language or motor) or the Bayley-III measure (e.g., over the Denver-II in Colombia 2).

**Table A3a: DAZ** in children under 48 months correlated with IQ in Middle Childhood (>4 to 9 years) and Adolescence (>9 to 18 years)

| | Time 1 | Time 2: Middle Childhood | | | | | Time 2: Adolescence | | | |
|---|---|---|---|---|---|---|---|---|---|---|
| **DAZ** | **Ages (months)** | **Ages (years; months)[d]** | **N** | **IQ[a]** | **Ravens[c]** | **PPVT** | **Ages (years; months)[d]** | **N** | **IQ[b]** | **PPVT** |
| Bangladesh | 18 | 5;3-5;8 | 1599 | **0·376** | | | | | | |
| Brazil 1 | 5-11 | | | | | | 18;6 | 1151 | **0·172** | |
| | 11-19 | | | | | | 18;6 | 1117 | **0·187** | |
| Chile 1 | 6 | 5;6-5;8 | 71 | **0·381** | | | | | | |
| | 12 | 5;6-5;8 | 834 | **0·194** | | | | | | |
| | 18 | 5;6-5;8 | 196 | 0·167 | | | | | | |
| Chile 2 | 24-35 | 4;1-5;2 | 2761 | | | **0·241** | | | | |
| | 36-47 | 5;1-6;2 | 2700 | | | **0·391** | | | | |
| Colombia 1 | 10-26 | 4;6-5;8 | 608 | | | **0·368** | | | | |
| | 28-45 | 4;6-5;8 | 561 | | | **0·521** | | | | |
| Colombia 2 | 6-17 | 6;0-6;11 | 280 | 0·126 | | 0·008 | | | | |
| | 18-29 | 6;10-7;11 | 336 | **0·424** | | **0·357** | | | | |
| | 30-42 | 7;10-8;11 | 324 | **0·437** | | **0·391** | | | | |
| Ecuador | 0-11 | 5;7-6;7 | 158 | | | 0·069 | 9;2-10;2 | 166 | | 0·070 |
| | 12-23 | 6;8-7;8 | 240 | | | 0·029 | 10;2-11;3 | 246 | | 0·122 |
| | 24-35 | 7;7-8;8 | 230 | | | 0·131 | 11;1-12;2 | 233 | | **0·226** |

| DAZ | Time 1 | Time 2: Middle Childhood | | | | | Time 2: Adolescence | | | |
|---|---|---|---|---|---|---|---|---|---|---|
| | **Ages (months)** | **Ages (years; months)[d]** | **N** | **IQ[a]** | **Ravens[c]** | **PPVT** | **Ages (years; months)[d]** | **N** | **IQ[b]** | **PPVT** |
| Ethiopia[e] | 11-12 | | | | | | 9;10-10;10 | 150 | | 0·113 |
| | 29-32 | | | | | | 9;10-10;10 | 369 | | **0·159** |
| | 41-44 | | | | | | 9;10-10;10 | 391 | | **0·163** |
| Jamaica 1 | 15 | 6;6-7;1 | 187 | **0·401** | | **0·323** | | | | |
| | 24 | 6;6-7;1 | 188 | **0·542** | | **0·533** | | | | |
| Jamaica 2 | 9-25 | 7;0-8;3 | 154 | **0·372** | **0·240** | **0·389** | 16;9-18;0 | 126 | **0·363** | |
| | 21-37 | 7;0-8;3 | 154 | **0·506** | **0·287** | **0·537** | 16;9-18;0 | 126 | **0·562** | |
| | 33-48 | 7;0-8;3 | 135 | **0·501** | **0·455** | **0·476** | 16;9-18;0 | 109 | **0·543** | |
| Madagascar | 34-42 | 6;10-7;10 | 174 | 0·172 | | 0·165 | | | | |
| Netherlands 1 | 0-11 | 4;7-6;1 | 2662 | **0·074** | | | | | | |
| | 12-23 | 4;7-6;1 | 1366 | **0·149** | | | | | | |
| | 24-32 | 4;7-6;1 | 396 | **0·287** | | | | | | |
| South Africa[f] | 6 | 4;10-5;6 & 7;0-8;6 | 196 | 0·183 | 0·052 | | | | | |
| | 12 | 4;10-5;6 & 7;0-8;6 | 155 | 0·115 | 0·059 | | | | | |
| | 24 | 4;10-5;6 & 7;0-8;6 | 935 | **0·104** | 0·053 | | | | | |

Differences significant <0·05 are in bold

[a]IQ measures at Time 2: middle childhood are WPPSI (Bangladesh, Chile 1, Jamaica 1), WISC-V (Colombia 2), SB-4 (Jamaica 2), SB-5 (Madagascar), UKKI (Netherlands 1) and Denver-II (South Africa)

[b]IQ measures at Time 2: adolescence are WAIS (Brazil 1, Jamaica 2)

[c]Raven's are the Raven's Progressive Matrices (Jamaica 2) and Raven's Coloured Progressive Matrices (South Africa)

[e]The PPVT in Ethiopia was a combined measure of versions 3 and 4

[f]South Africa had follow-up measures at 5 years (Denver-II) and 7 years (Raven's) at Time 2

**Table A3b:** Original* age-standardized measures in children under 48 months correlated with IQ in Middle Childhood (>4 to 9 years) and Adolescence (>9 to 18 years)

| | Time 1 | Time 2: Middle Childhood | | | | | Time 2: Adolescence | | | |
|---|---|---|---|---|---|---|---|---|---|---|
| **DAZ** | **Ages (months)** | **Ages (years; months)[d]** | **N** | **IQ[a]** | **Ravens[c]** | **PPVT** | **Ages (years; months)[d]** | **N** | **IQ[b]** | **PPVT** |
| Bangladesh | 18 | 5;3-5;8 | 1599 | **0·347** | | | | | | |
| Brazil 1 | 5-11 | | | | | | 18;6 | 1151 | **0·224** | |
| | 11-19 | | | | | | 18;6 | 1117 | **0·189** | |
| Chile 1 | 6 | 5;6-5;8 | 71 | **0·379** | | | | | | |
| | 12 | 5;6-5;8 | 834 | **0·214** | | | | | | |
| | 18 | 5;6-5;8 | 196 | **0·252** | | | | | | |
| Chile 2 | 24-35 | 4;1-5;2 | 2761 | | | **0·266** | | | | |
| | 36-47 | 5;1-6;2 | 2700 | | | **0·407** | | | | |
| Colombia 1 | 10-26 | 4;6-5;8 | 608 | | | **0·249** | | | | |
| | 28-45 | 4;6-5;8 | 561 | | | **0·447** | | | | |
| Colombia 2 | 6-17 | 6;0-6;11 | 280 | 0·137 | | 0·119 | | | | |
| | 18-29 | 6;10-7;11 | 336 | **0·394** | | **0·305** | | | | |
| | 30-42 | 7;10-8;11 | 324 | **0·469** | | **0·344** | | | | |
| Ecuador | 0-11 | 5;7-6;7 | 158 | | | 0·121 | 9;2-10;2 | 166 | | 0·051 |
| | 12-23 | 6;8-7;8 | 240 | | | 0·046 | 10;2-11;3 | 246 | | 0·145 |
| | 24-35 | 7;7-8;8 | 230 | | | 0·072 | 11;1-12;2 | 233 | | **0·204** |
| Ethiopia[d*] | 11-12 | | | | | | 9;10-10;10 | 150 | | 0·038 |
| | 29-32 | | | | | | 9;10-10;10 | 369 | | **0·196** |
| | 41-44 | | | | | | 9;10-10;10 | 391 | | **0·162** |
| Jamaica 1 | 15 | 6;6-7;1 | 187 | **0·401** | | **0·313** | | | | |
| | 24 | 6;6-7;1 | 188 | **0·498** | | **0·439** | | | | |
| Jamaica 2 | 9-25 | 7;0-8;3 | 154 | **0·328** | 0·033 | 0·138 | 16;9-18;0 | 126 | 0·211 | |
| | 21-37 | 7;0-8;3 | 154 | **0·525** | **0·289** | **0·535** | 16;9-18;0 | 126 | **0·543** | |
| | 33-48 | 7;0-8;3 | 135 | **0·571** | **0·443** | **0·535** | 16;9-18;0 | 109 | **0·593** | |

| | Time 1 | Time 2: Middle Childhood | | | | | Time 2: Adolescence | | | |
|---|---|---|---|---|---|---|---|---|---|---|
| **DAZ** | **Ages (months)** | **Ages (years; months)[d]** | **N** | **IQ[a]** | **Ravens[c]** | **PPVT** | **Ages (years; months)[d]** | **N** | **IQ[b]** | **PPVT** |
| Madagascar | 34-42 | 6;10-7;10 | 174 | 0·218 | | 0·165 | | | | |
| Netherlands 1 | 0-11 | 4;7-6;1 | 2655 | **0·076** | | | | | | |
| | 12-23 | 4;7-6;1 | 1361 | **0·166** | | | | | | |
| | 24-32 | 4;7-6;1 | 394 | **0·289** | | | | | | |
| South Africa[e] | 6 | 4;10-5;6 & 7;0-8;6 | 196 | 0·122 | -0·041 | | | | | |
| | 12 | 4;10-5;6 & 7;0-8;6 | 155 | 0·168 | 0·077 | | | | | |
| | 24 | 4;10-5;6 & 7;0-8;6 | 935 | 0·142 | 0·051 | | | | | |

Differences significant <0·05 are in bold

*For the original instruments, Bayley-I and Bayley-II, we used the MDI in the correlations (Bangladesh, Chile 1, South Africa); for Bayley III, we used the measure from the cognition domain (Colombia 1, Colombia 2, Ethiopia).

[a]IQ Measures at Time 2: middle childhood are WPPSI (Bangladesh, Chile 1, Jamaica 1), WISC-V (Colombia 2), SB-4 (Jamaica 2), SB-5 (Madagascar), UKKI (Netherlands 1) and Denver-II (South Africa)

[b]IQ measures at Time 2: adolescence are WAIS (Brazil 1, Jamaica 2)

[c]Ravens are the Ravens Progressive Matrices[13] (Jamaica 2) and Ravens Coloured Progressive Matrices[14] (South Africa)

[d]Age in years rounded to nearest half year

[e]The PPVT in Ethiopia was a combined measure of versions 3 and 4

[f]South Africa had follow-up measures at 5 years (Denver-II) and 7 years (Ravens) at Time 2
